# XiP: a computational environment to create, extend and share workflows

**DOI:** 10.1093/bioinformatics/bts630

**Published:** 2012-10-25

**Authors:** Masao Nagasaki, André Fujita, Yayoi Sekiya, Ayumu Saito, Emi Ikeda, Chen Li, Satoru Miyano

**Affiliations:** ^1^Department of Integrative Genomics, Tohoku Medical Megabank Organization, Tohoku University, Japan, ^2^Department of Computer Science, Institute of Mathematics and Statistics, University of São Paulo, Brazil and ^3^Human Genome Center, Institute of Medical Science, University of Tokyo, Japan

## Abstract

XiP (eXtensible integrative Pipeline) is a flexible, editable and modular environment with a user-friendly interface that does not require previous advanced programming skills to run, construct and edit workflows. XiP allows the construction of workflows by linking components written in both R and Java, the analysis of high-throughput data in grid engine systems and also the development of customized pipelines that can be encapsulated in a package and distributed. XiP already comes with several ready-to-use pipeline flows for the most common genomic and transcriptomic analysis and ∼300 computational components.

**Availability:** XiP is open source, freely available under the Lesser General Public License (LGPL) and can be downloaded from http://xip.hgc.jp.

**Contact:** nagasaki@megabank.tohoku.ac.jp

## 1 INTRODUCTION

Large-scale sequencing and microarray technologies are high-throughput methodologies that generate huge genomic and transcriptomic data that must be processed in a multi-step fashion. Usually, it is carried out by several distinct programs that are interconnected in a specific order, forming a workflow process, namely pipeline (Durham *et al.*, 2004; Fujita *et al.*, 2007). For example, a simple workflow to investigate genes potentially related to cancer might begin with microarray image analysis, normalization, statistical tests to identify differentially expressed genes between the normal and the tumor tissues followed by a multiple test *P*-value correction.

Shah *et al.* (2004) have described that pipelines must satisfy at least three characteristics: (i) *flexibility*: a software can be used to analyze different data sets that may require different analysis tools; (ii) *integrability*: a system should provide the framework to facilitate data integration of analysis results from different tools; and (iii) *extensibility*: a system needs to allow for the inclusion of new tools in a modular fashion.

In addition to these characteristics that are actually necessary to a pipeline, we believe that the portability with grid engines and the interoperability with pre-existing systems are also important in this new era of generation of high-throughput data. The portability with grid engines makes possible to run heavy routines in supercomputers (hundreds of cores) in an easy manner while the interoperability allows the use of workflows constructed under different platforms.

To facilitate the construction of workflows, we present XiP (eXtensible integrative Pipeline), a free [under the Lesser General Public License (LGPL)] and easy-to-use environment designed to integrate the state-of-the-art computational methods and to satisfy researchers’ need in multi-collaborative projects.

## 2 IMPLEMENTATION

XiP was entirely developed in Java and runs at the client’s machine via the Java Web Start technology. In other words, XiP runs in the majority of operating systems, requiring only a pre-installation of the Java Runtime Environment (JRE version ≥1.6) at the client’s machine. If JRE is not installed, the installation package asks for the permission to install JRE. Although XiP was originally designed to run via the Web, it can also be installed in local machines.

XiP already comes with ∼300 components, where each component represents one computational algorithm (e.g. Support Vector Machine, k-means, *t*-test, etc). XiP also recognizes components written in R (R Development Core Team, 2011), one of the most popular statistical programming languages in Bioinformatics.

For data input, XiP accepts any Java and R basic data structures, Cell System Markup Language (CSML) (Nagasaki *et al.*, 2010), Cell System Ontology (CSO) (Jeong *et al.*, 2007), Cell System Markup Language Data Base (CSMLDB) and CSODB formats.

The complete list of components that comes with XiP (∼300 components), tutorials, documentation and some example pipelines are available at the XiP project webpage (http://xip.hgc.jp).

## 3 RESULTS AND DISCUSSIONS

With the advances in the generation of high-throughput data and the development of large-scale projects, which involve dozens of labs around the world, computational pipelines become crucial and indispensable, especially when the same protocol must be carried out in different laboratories to guarantee both reproducibility and quality.

**Fig. 1.**
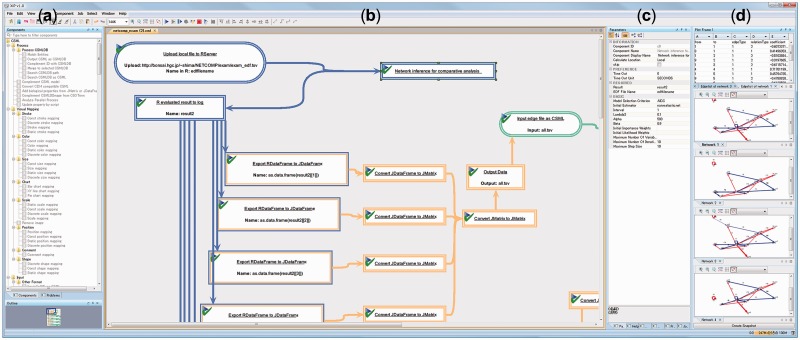
Screenshot of a XiP graphical interface and a regulatory network estimation pipeline. (**a**) The list of components; (**b**) the pipeline schema; (**c**) the parameters for each component of the pipeline; (**d**) the output, i.e. the estimated regulatory network

The construction of a computational pipeline under the XiP platform does not require advanced computer-programming skills. At Figure 1a, there is a list of components and a tool to search for a specific algorithm. To build a workflow, the user clicks on the component of interest and drags and drops on the canvas (Fig. 1b). The order of analysis in the pipeline is set according to the order of the components defined by the arrows of the workflow (Fig. 1b). The tail of the arrow represents from which component the data comes, and the head points to the next analysis step. In other words, the direction of the arrows indicates the data flow. The parameters of each component can be easily set up by using the graphical interface illustrated on Figure 1c, and the results are visualized in separate windows as shown in Figure 1d.

XiP satisfies the three essential characteristics for a pipeline platform and also the fourth (portability to grid engines) and fifth (interoperability) described in Section 1 as follows:

1. *Flexibility*: The specific requirements of a research project make it difficult to use a pipeline designed for a particular data set for analysis of another data set. As a result, two different pipelines must be constructed, both sharing several common components. However, notice that it is not necessary to reconstruct the entire pipeline, but only the different parts. As the pipelines constructed by XiP are modular, i.e. the pipelines are composed of an ordered sequence of components, one must replace only the different components to adapt the pipeline to a new data set.
2. *Integrability*: Components written in both R and Java programming languages run on XiP. Internally, XiP translates the R data structures into Java structures, thus allowing the connection of packages available at the R webpage (http://www.r-project.org) and the BioJava project (Holland *et al.*, 2008).
3. *Extensibility*: R and Java functions developed by different groups can automatically be translated to a XiP component and included in the platform. Therefore, XiP can be customized and extended with several components depending on the user’s necessities.
4. *Portability to grid engine*: The analysis of large amounts of data generated by the new technological approaches in molecular biology requires high-performance computational resources. The XiP platform allows the construction of pipelines that use grid engines to parallelize computational jobs. To run a parallel job, the user must set up a cluster (server) with several cores and log in to this remote server. The integration with grid engines makes XiP suitable for individual researchers with modest data sizes as well as for big collaborative projects with large amounts of data.
5. *Interoperability*: Owing to several different pipeline platforms available in the literature such as Pegasus (Deelman *et al.*, 2004), Kepler (Altintas *et al.*, 2004) and Galaxy (Goecks *et al.*, 2010), each one with unique advantages, it would be interesting whether one platform could integrate components developed in different environments. XiP satisfies the interoperability by converting the components developed in the Galaxy platform (Goecks *et al.*, 2010) to an XiP component. Interoperability with other systems is under development.

Once a pipeline is constructed, it can be distributed in both a XiP XML format and a *jar* file. The XiP XML is a markup language that stores the pipeline structure, i.e. the information about how the components are interconnected. The *jar* format is a closed stand-alone package that runs in a computational environment without XiP. Some examples of pipelines freely available designed in the XiP platform are the DA1.0 (Koh *et al.*, 2010) and the CSO validator (Jeong *et al.*, 2011). Other examples can be found at the XiP webpage.

Summing up, the main advantages of XiP are (i) it satisfies all the five characteristics for a pipeline platform; (ii) components developed in the Galaxy platform can be converted to XiP components; and (iii) stand-alone packages created under XiP can be run outside the XiP environment.

The entire code is open and we encourage researchers to contribute with novel functionalities for the XiP platform.

## References

[bts630-B1] Altintas I (2004). Kepler: an extensible system for design and execution of scientific
workflows. Proceedings of the 16th International Conference on Scientific and Statistical
Database Management.

[bts630-B2] Deelman E (2004). Pegasus: mapping scientific workflows onto the grid. Lect. Notes Comput. Sci..

[bts630-B3] Durham AM (2004). EGene: a configurable pipeline generation system for automated sequence
analysis. Bioinformatics.

[bts630-B4] Fujita A (2007). GEDI: a user-friendly toolbox for analysis of large-scale gene expression
data. BMC Bioinformatics.

[bts630-B5] Goecks J (2010). Galaxy: a comprehensive approach for supporting accessible, reproducible,
and transparent computational research in the life sciences. Genome Biol..

[bts630-B6] Jeong E (2007). Cell system ontology: representation for modeling, visualizing and
simulating biological pathways. In Silico Biol..

[bts630-B7] Jeong E (2011). CSO validator: improving manual curation workflow for biological
pathways. Bioinformatics.

[bts630-B8] Nagasaki M (2010). Cell Illustrator 4.0: a computational platform for systems
biology. In Silico Biol..

[bts630-B9] Holland RCG (2008). BioJava: an open-source framework for bioinformatics. Bioinformatics.

[bts630-B10] Koh CH (2010). DA 1.0: a parameter estimation of biological pathways using data
assimilation approach. Bioinformatics.

[bts630-B11] R Development Core Team (2011). R: a language and environment for statistical computing.

[bts630-B12] Shah SP (2004). Pegasys: software for executing and integrating analyses of biological
sequences. BMC Bioinformatics.

